# Identification of the Cytotoxic Transglutaminase from *Mycobacterium* spp. That Is Involved in RIPK1 Activation

**DOI:** 10.3390/molecules30102251

**Published:** 2025-05-21

**Authors:** Xinting Zhang, Yikai Zhang, Xiao Feng, Yueying Wang, Si-Shang Li, Mei-Yi Yan, Yi-Cheng Sun, Qi Jin, Feng Jiang

**Affiliations:** NHC Key Laboratory of Systems Biology of Pathogens, Key Laboratory of Pathogen Infection Prevention and Control (Ministry of Education), State Key Laboratory of Respiratory Health and Multimorbidity, National Institute of Pathogen Biology and Center for Tuberculosis Research, Chinese Academy of Medical Sciences & Peking Union Medical College, Beijing 102629, China; zxtwangyi2021@163.com (X.Z.); yikaizhang@yeah.net (Y.Z.); fengxiao931209@163.com (X.F.); wyy3651@163.com (Y.W.); liciera@163.com (S.-S.L.); ymy0106@ipbcams.ac.cn (M.-Y.Y.); sunyc@ipbcams.ac.cn (Y.-C.S.)

**Keywords:** *Mycobacterium* spp., MmTG, RIPK1, cytotoxicity, transglutaminase

## Abstract

Although the global incidence of tuberculosis has declined in recent years, tuberculosis remains a major global public health challenge. The *Mycobacterium tuberculosis* complex (MTBC) including *M. tuberculosis*, *M. bovis*, *M. microti*, etc., is the deadliest *Mycobacterium* spp. that needs more attention. Research on *M. microti* is significant as it is a zoonotic pathogen that can spread between animals and humans. By exploring the function of a transglutaminase in *M. microti* (MmTG), which is widely distributed in *Mycobacterium* and other species, a potential cytotoxic effector has been characterized. MmTG inhibits cell proliferation by inducing the phosphorylation of RIPK1 (receptor-interacting serine/threonine-protein kinase 1) and the Cys159 of MmTG is the highly conserved residue related to its cytotoxicity. Understanding MmTG and its homologs can provide more insights into the pathogenic mechanisms of mycobacteria and contribute to the development of more effective therapeutic strategies against mycobacterial infections.

## 1. Introduction

As one of the most significant infectious diseases, tuberculosis (TB) remains a major public health concern, especially for low- and middle-income countries. In 2023, there were 8.2 million new confirmed cases of tuberculosis globally, a number that continues to rise compared to previous years. Ending the global TB epidemic remains a distant goal [1]. The *Mycobacterium tuberculosis* complex (MTBC) comprises a group of highly related mycobacteria, including *M. tuberculosis*, *M. bovis*, *M. microti*, and other species [2], which infect a wide range of hosts, primarily causing tuberculosis in various mammals with varying degrees of severity. Due to the diversity of the pathogenic mechanisms of the MTBC, its strong ability to evade host immunity, its high genetic variability, and the complexity of acquired drug resistance [3,4], the clinical diagnosis and treatment of tuberculosis face numerous challenges. These factors not only complicate patient management but also impose a substantial economic burden on patients and their families.

TB, caused by *M. tuberculosis*, is primarily transmitted via the respiratory tract. While it mainly presents as pulmonary tuberculosis, it can also disseminate to multiple organs and tissues throughout the body, resulting in extrapulmonary manifestations such as skeletal tuberculosis and other forms of localized or systemic TB [5]. Moreover, *M. bovis*, the primary causative agent of bovine tuberculosis, is also considered to be associated with human disease. Humans may become infected with *M. bovis* through direct contact with livestock or through the consumption of unpasteurized dairy products and undercooked meat. Such cases are particularly prevalent in certain developing countries [6]. Thus, for human health and TB prevention, in addition to focusing on human pathogens, effectively controlling the spread of animal-hosted pathogens can directly impact the prevalence of human tuberculosis. Tuberculosis was first identified in voles in 1948 and was subsequently designated *M. microti*, later classified as a member of the MTBC [7]. It primarily infects voles but has also demonstrated a broad host range, exhibiting pathogenicity in animals such as goats, badgers, wild boars, and dogs, as well as in humans [8,9]. As a zoonotic pathogen, *M. microti* exhibits slow growth under in vitro conditions and possesses biochemical characteristics that are difficult to differentiate from those of other members of the MTBC. In addition, its pathogenic mechanisms in humans remain inadequately characterized, thereby presenting significant challenges for clinical diagnosis and treatment [10].

Transglutaminase (TGase) is an enzyme that catalyzes acyl transfer reactions, which can be classified based on their origin in animal-derived TGases, plant-derived TGases, and microbial transglutaminases (mTGs) [11,12]. Animal-derived TGases have been extensively studied, among which transglutaminase 2 (TG2) is widely distributed and multifunctional. TG2 plays pivotal roles in gene expression, signal transduction, immunity, and inflammation. It participates in diverse cellular metabolic processes, including transamidation, functioning as a Gα signaling protein, protein disulfide isomerase (PDI), protein kinase, and scaffold protein. While in the nucleus, it modifies histones and transcription factors [13]. However, the biological roles of mTGs in the MTBC remain poorly understood. TGases may play important roles in mycobacterial pathogenicity, potentially functioning as toxins, and they share a conserved Cys-His-Asp in the primary enzymatic active site [14,15]. The *Photorhabdus* virulence cassette (PVC), an extracellular contractile injection secretion system, possesses a syringe-like structure capable of delivering effector proteins (e.g., toxin) into host cells [16], which serves as an excellent tool for studying bacterial cytotoxic effects. We have previously utilized the PVC to characterize the activity of potential bacterial toxins and to investigate their interactions with host cells [17].

Herein, we characterized a gene, namely PLV44373.1, in *Mycobacterium tuberculosis* variant *microti* OV254 (*M. microti* transglutaminase, MmTG), which encodes a transglutaminase. Through transient expression, PVC-mediated delivery into J774A.1 cells, and bacterial infection of macrophages, we assessed the cytotoxicity and identified a conserved active site of MmTG. Bioinformatics analysis revealed that TGase is widely distributed and highly conserved among mycobacterial species. Moreover, the homologous protein in *M. abscessus* also exhibits cytotoxicity toward eukaryotic cells and induces RIPK1 phosphorylation. This study contributes to the identification of potential mycobacterial toxins and provides new insights into their pathogenic mechanisms.

## 2. Results

### 2.1. MmTG Inhibits Cell Proliferation

**Figure 1:**
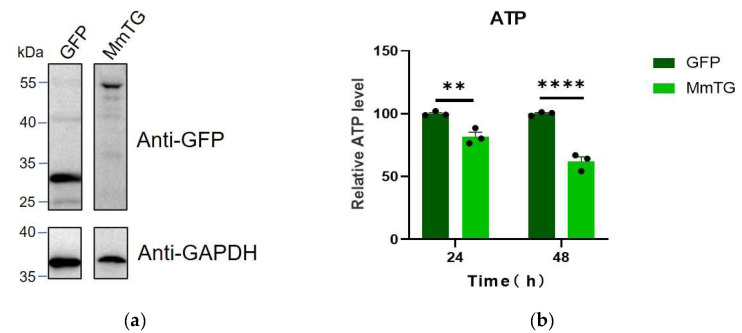
The MmTG protein from *M. microti* exhibits cytotoxic properties. (**a**) Western blot analysis was used to detect the transient expression of MmTG in HEK293T cells. GAPDH was detected as a loading control. From the same blot, lanes were rearranged. (**b**) Cell viability of HEK293T cells was assessed using ATP content analysis at 24 h and 48 h post-transient expression of MmTG, respectively. (**) *p* < 0.01; (****) *p* < 0.0001.

We initially constructed the eukaryotic expression vector pEGFP-C1-MmTG for MmTG and transiently expressed it in HEK293T cells. The expression of both GFP and the GFP-MmTG fusion proteins was confirmed by Western blot analysis (Figure 1a). After 24 and 48 h of GFP-MmTG expression, significant differences in cell viability were observed compared to the control group (Figure 1b). Given that ATP serves as the direct energy source for cells and is closely associated with cell viability, we assessed cell viability through ATP detection. The results demonstrated that the transient expression of the MmTG protein exerted cytotoxic effects on HEK293T cells, potentially acting as a cytotoxic effector molecule of *M. microti*.

### 2.2. Infection Assay of M. smegmatis

**Figure 2:**
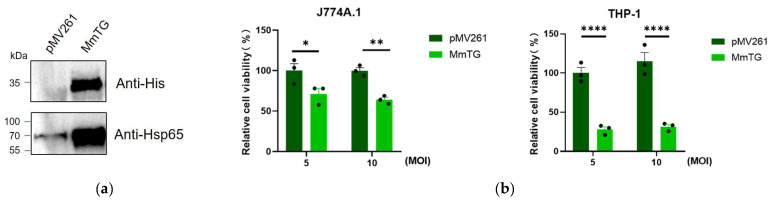
Infection of eukaryotic macrophages by *M. smegmatis* expressing MmTG. (**a**) MmTG was expressed in *M. smegmatis* and detected using Western blotting. Cell lysates of *M. smegmatis*-expressed MmTG were subjected to Western blotting to determine the expression of His-tagged MmTG protein in *M. smegmatis* by anti-His antibody. pMV261 is an empty vector and serves as a negative control. Hsp65 was detected as a loading control. (**b**) Cell viability of J774A.1 and THP-1 cells were assessed using the CCK-8 analysis after infection with *M. smegmatis* expressing MmTG for 24 h. (*) *p* < 0.05; (**) *p* < 0.01; (****) *p* < 0.0001.

To further explore the toxic effects of MmTG, we performed *M. smegmatis* infection assays in macrophages followed by cell viability detection using a Cell Counting Kit-8 (CCK-8) analysis. As is shown in Figure 2a, we successfully expressed MmTG in *M. smegmatis.* Infection of J774A.1 and THP-1 cells with recombinant *M. smegmatis* for 24 h displayed a significant reduction in cell viability (Figure 2b), with a more pronounced decrease observed in human THP-1 cells.

### 2.3. Cys159 Is Essential for Function

**Figure 3:**
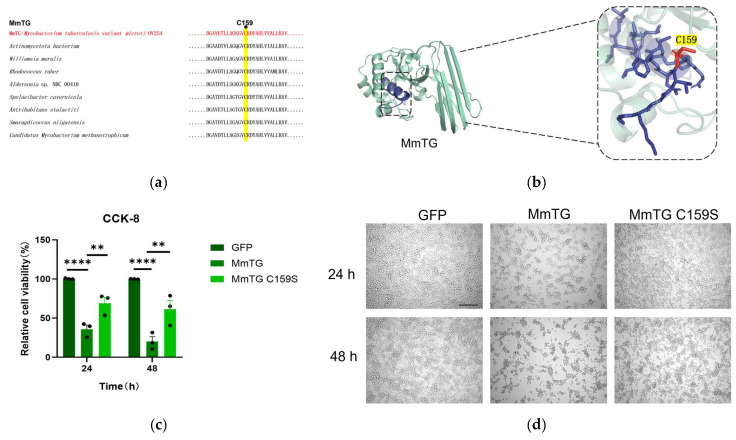
The cysteine at the active site of MmTG is highly conserved and is essential for MmTG exerting cytotoxicity. (**a**) The active site C159 of MmTG was highly conserved in different genera. (**b**) The 3D model of MmTG module. Sequences of the MmTG were analyzed by AlphaFold3 and the C159 key residue was highlighted. (**c**) Cell viability of HEK293T cells was assessed using the CCK-8 analysis at 24 h and 48 h post-transient expression of MmTG and C159S, respectively. (**) *p* < 0.01; (****) *p* < 0.0001. (**d**) Cell morphology following 24 h and 48 h transient expression of MmTG or C159S in HEK293T cells. Scale bars, 200 μm.

The catalytic triad Cys-His-Asp is highly conserved in TGases, with particular emphasis on Cys159, which plays a crucial role in both evolutionary history and biochemical activity (Figure 3a, Table S1). And, structural prediction of MmTG via AlphaFold3 determined the tertiary architecture: four α-helices and two β-sheets interconnected by loop regions (Figure 3b), which exhibit remarkable similarity to canonical transglutaminase folds. Site-directed mutagenesis was performed and transiently expressed the mutant (C159S) in HEK293T cells. The cell viability assay demonstrated that the eukaryotic toxicity of the mutant was markedly diminished in comparison to the wild-type protein (Figure 3c). There was a distinct morphological difference between mutant and wild-type-transfected cells at both time points (Figure 3d).

### 2.4. MmTG Is Loaded in the PVC for the Direct Killing of Murine Macrophages

**Figure 4:**
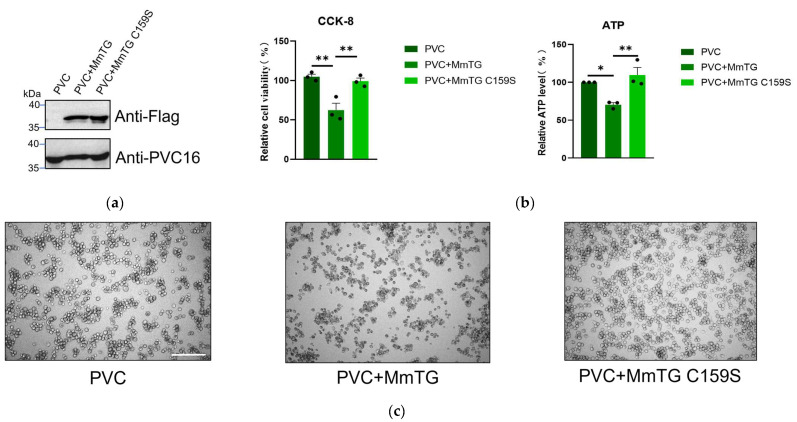
MmTG can be loaded in PVC for direct killing of murine macrophages. (**a**) Loading of MmTG was detected by Western blot analysis. Pvc16 was used as a loading control. (**b**) Cell viability of J774A.1 cells was assessed using the CCK-8 (**left**) and ATP content analysis (**right**) at 24 h post-delivery of MmTG or C159S mutant by PVC. (*) *p* < 0.05; (**) *p* < 0.01. (**c**) Cell morphology of J774A.1 cells after treatment with PVC+MmTG or PVC+MmTG C159S for 24 h. Scale bars, 200 μm.

To simulate the biological process of protein action on host cells via the secretion system, the PVC, a successful delivery vehicle identified in our prior research, was used to package and deliver both wild-type and mutant proteins into macrophages (Figure 4a), thereby validating the functional significance of the MmTG. PVC-delivered mutants exhibited markedly attenuated cytotoxicity, with observable differences in cells at 24 h post-delivery (Figure 4b,c). These results confirmed that MmTG acts as a cytotoxic effector of *M. microti*, and the Cys159 has contributed an essential role to the cytotoxic effects.

### 2.5. TGases Are Widely Distributed and Induce the Phosphorylation of RIPK1

**Figure 5:**
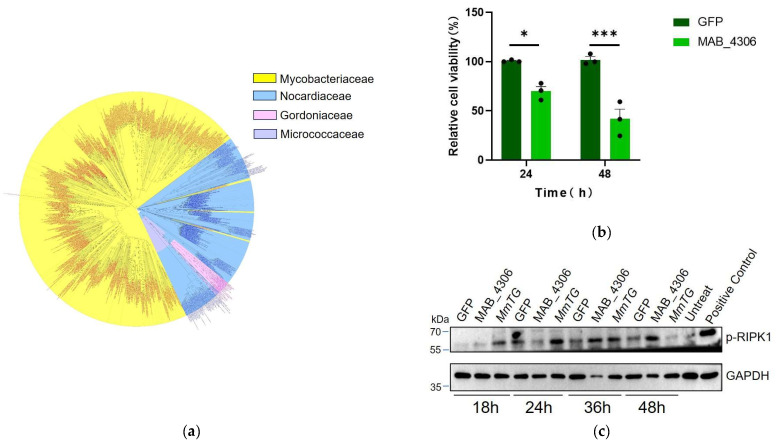
Transglutaminase is widespread and conserved in mycobacteria. (**a**) To analyze the evolutionary relationship of transglutaminase in mycobacteria, a phylogenetic tree was constructed by Neighbor-Joining method. (**b**) Cell viability of HEK293T cells was assessed using the CCK-8 at 24 h and 48 h post-transient expression of MAB_4306, the homologous protein of MmTG in *M. abscessus*. (*) *p* < 0.05; (***) *p* < 0.001. (**c**) After transfection with MmTG and MAB_4306, the expression level of p-RIPK1 in HEK293T cells was detected by Western blot analysis. Positive control: cells were treated with Necroptosis Inducer Kit, which contains TNF-α, SM-164, and Z-VAD-FMK for 4 h.

TGases are widely distributed in 1000 bacterial cells, including *Mycobacterium*, *Nocardia*, *Gordonia*, and *Micrococcus*, accounting for nearly three-quarters of all identified homologs in *Mycobacterium* with high sequence conservation (Figure 5a). The transient expression of MAB_4306, a homologous protein of MmTG in *M. abscessus*, in HEK293T cells for 24 and 48 h, led to a significant reduction in cell viability (Figure 5b). The PVC was also employed to deliver the homologous protein MAB_4306 to J774A.1 cells (Figure S1a). A significant decrease in cell viability was also detected (Figure S1b), and the highly conserved Cys159 related to its cytotoxicity (Figure S1c–f). Moreover, both TGases can induce the phosphorylation of RIPK1 at different time points (MmTG activates the RIPK1 at 18 h and MAB_4306 at 36 h) (Figure 5c). As a pivotal mediator of both cell death and inflammatory responses, RIPK1 activation has been demonstrated to play a critical role in both apoptosis and necroptosis, potentially accounting for the cell death observed in this study.

## 3. Discussion

The MTBC comprises a group of zoonotic acid-fast bacilli that typically cause pulmonary or extrapulmonary tuberculosis in hosts through infection by a single species. Typical multidrug-resistant NTM, *M. abscessus*, and MTBC pathogens, namely *M. bovis*, and *M. microti.*, can be transmitted to humans via environmental sources, contaminated water, wildlife, livestock, or unpasteurized animal products [17,18], representing a significant public health threat. Human *M. microti* infections has been primarily reported in European countries [19,20]; notably, *M. microti* can cause clinical disease even in immunocompetent individuals [21,22], and the infections typically occur through contact with wildlife or consumption of contaminated water. Research on *M. microti* has focused on epidemiology [23,24,25], infection characteristics [8], and molecular diagnostics [26,27,28]; however, the exploration of its pathogenic mechanisms or host interactions is limited.

TGases are a widely distributed in animals, plants, and microbes. Studies on mTGs mainly focused on structural characterization, protein expression/purification, and applications in food processing, but its interaction mechanisms with hosts in terms of microbial pathogens have not yet been determined. This study firstly presents the evidence suggesting that TGase may play a crucial role in mycobacterial pathogenesis by mediating cytotoxicity through the regulation of cell death pathways. Overexpression of MmTG significantly reduced the cell viability of HEK293T cells. Macrophages serve as the primary host cells for *M. tuberculosis* infection in vivo and represent crucial immune effector cells mediating bacterial growth control [29]. To further evaluate the cytotoxic effects of MmTG, macrophage infection experiments by *M. smegmatis* have been performed and have assessed the cellular viability of J774A.1 and THP-1 cells. THP-1 cells exhibited lower viability compared to J774A.1 cells, which may be attributed to two factors: firstly, this bacterial protein demonstrates higher receptor affinity in human cells compared to murine cells. Secondly, the differential immune responses elicited by *M. smegmatis* infection across cell lines may impact cellular viability. Previous studies have shown that THP-1 cells predominantly eliminate infected cells through apoptosis [30,31,32], suggesting that this bacterial protein may exert its cytotoxic effects by modulating apoptotic pathways.

As an extracellular contractile injection secretion system, the PVC utilizes its syringe-like structure to deliver cargo proteins (effectors) into host organisms [33]. Our previous research revealed that the PVC gene cluster encodes several proteins with demonstrated eukaryotic cytotoxicity, and identified the N-terminal signal peptide sequences from these effectors (Pdp1 and Pnf). When these signal peptides were fused to heterologous proteins (e.g., reporter genes or antitumor proteins), they facilitated the loading of these proteins onto the PVC and subsequent delivery into target cells [34]. These findings establish the PVC delivery vector as a powerful tool for investigating the pathogenic mechanisms of the *M. microti* cytotoxic effector. MmTG has been delivered into J774A.1 cells mediated by the PVC, and consistent with the transfection results, there was a significant decrease in cell viability. The catalytic triad Cys-His-Asp in TGases plays a critical role in biochemical activity, and Cys159 is important in the catalytic functions of MmTG, for there was a substantially attenuated eukaryotic toxicity in the C159S compared with the wild type, which has been determined through transfection and PVC-delivery assays. However, a previous study has indicated that some mTGs typically exist in the bacterial cytoplasm as zymogens and are activated by proteolytic cleavage on the bacterial cell wall by proteases produced by the bacteria themselves, thereby acquiring enzymatic activity [35]. In this study, it remains unclear whether the MmTG protein obtains its enzymatic activity through a similar activation mechanism. This question warrants further investigation in future studies.

In addition, bioinformatics analysis demonstrated that mTGs are widely distributed in *Mycobacterium*; significantly, it has also been found in clinically human pathogens, such as *M. abscessus*, *M. interjectum*, *M. marseillense*, *M. parmense*, etc., (Figure S2, Table S2). We focused on the homolog in *M. abscessus*, and a similar decrease in cell viability was detected. The toxic mechanisms of the two mTGs were investigated for inducing eukaryotic cell death. It was revealed that both proteins can activate RIPK1 and induce its phosphorylation upon interacting with cells. RIPK1 is a serine/threonine protein kinase that plays an important role in apoptosis, necroptosis, and inflammatory signaling pathways. Currently, RIPK1has emerged as a potential therapeutic target for diseases such as Alzheimer’s disease, multiple sclerosis, stroke, and traumatic brain injury [36]. Nevertheless, the mechanisms by which these two proteins induce RIPK1 phosphorylation and the specific cell death pathways involved remain unclear, as well as the potential involvement of interacting proteins or molecules in the cell death process, which need further validation.

## 4. Materials and Methods

### 4.1. Bacterial and Cell Culture

#### 4.1.1. Bacterial Culture

*E. coli* strains were cultured in LB broth at 37 °C. The *E. coli* strain DH5α was employed for DNA manipulation, while *E. coli* EPI300 was utilized for PVC purification. *Mycobacterium smegmatis* mc2155 and the recombinant strains Ms_Vec and Ms_MmTG were cultivated in Middlebrook 7H9 broth medium at 37 °C with shaking at 220 rpm. Antibiotics were used at the following concentrations: ampicillin, 100 μg/mL; tetracycline, 10 μg/mL; kanamycin, 25 μg/mL; gentamycin, 20 μg/mL; chloramphenicol, 25 μg/mL.

#### 4.1.2. Cell Culture

The murine macrophage-like cell line J774A.1 and human embryonic kidney 293T cells HEK293T cells were cultured in Dulbecco’s Modified Eagle Medium (DMEM, Thermo Fisher Scientific, New York, NY, USA, C11995500BT) supplemented with 10% fetal bovine serum (FBS, Thermo Fisher Scientific, A5669701), 1×Penicillin–Streptomycin (MedChemExpress, New Jersey, NJ, USA, 100 μg/mL streptomycin, and 100 U/mL penicillin, HY-K1006). The human monocytic leukemia cell line THP-1 cell line was cultured in RPMI 1640 medium (Thermo Fisher Scientific, C11875500BT) containing 10% FBS, 1×Penicillin–Streptomycin. All cells were maintained in a humidified incubator at 37 °C with 5% CO_2_.

### 4.2. Plasmid Construction and DNA Manipulation

In this study, pEGFP-C1 was employed for the transient expression of MmTG in eukaryotic cells, pMV261 was utilized for the expression of MmTG in *Mycobacterium smegmatis*, and pBBRN-pdp1N50 served as the vector for the expression of PVC cargo proteins. The recombinant plasmid pRK404-PVC, harboring 16 structural genes of the PVC, and the recombinant plasmid pBR322-LysR, containing regulatory genes essential for PVC assembly, were used to facilitate the assembly of PVCs. The *MmTG* gene sequence was synthesized with a 5′ end incorporating the forward homologous arm of the pMV261 plasmid and a BamHI restriction site, and a 3′ end including the reverse homologous arm of the pMV261 plasmid, a HindIII restriction site, and a C-terminal 6×His tag. For eukaryotic transient expression, primers were designed with the forward homologous arm of the pEGFP-C1 plasmid and a BglII restriction site at the 5′ end, and the reverse homologous arm of the pEGFP-C1 plasmid and a SalI restriction site at the 3′ end. The PCR fragment obtained was double digested with BglII (Thermo Fisher Scientific, FD0084) and SalI (Thermo Fisher Scientific, FD0644) (Figure S3a) and ligated into the pEGFP-C1 vector, which had been double digested with BglII and SalI (Figure S3b), through homologous recombination. Primers were designed with the forward homologous arm of the pBBRN-pdp1N50 plasmid and a BamHI restriction site at the 5′ end, and the reverse homologous arm of the pBBRN-pdp1N50 plasmid and a HindIII restriction site at the 3′ end. The resulting PCR fragment was double digested with BamHI (Thermo Fisher Scientific, FD0054) and HindIII (Thermo Fisher Scientific, FD0505) (Figure S3a) and subsequently ligated into the pBBRN-pdp1N50 vector (Figure S3b), which had been double digested with the same enzymes, through homologous recombination for protein expression and loading in the PVC. Following double digestion with BamHI and HindIII, the synthesized fragment was ligated into the pMV261 vector, which had been similarly digested, enabling protein expression in *Mycobacterium smegmatis* (Figure S3c). The primers used in this step are shown in the Table S3.

### 4.3. Western Blot Analysis

The protein solution of the PVC for Western blot detection and the lysate of HEK293T cells were added with protein loading buffer and heated at 99 °C for 10 min. The *Mycobacterium smegmatis* that infected the cells was added with protein loading buffer and heated at 99 °C for 1 h. Then, the samples were separated through a 12% tris-glycine protein gel. The protein bands in the gel were transferred to a PVDF membrane (Millipore, Burlington, MA, USA, IPVH00010) using a Bio-Rad transfer apparatus. The membrane was blocked with 5% BSA in TBST for 1 h, and then incubated with the corresponding primary antibody at 4 °C overnight. If necessary, the membrane was further incubated with HRP-conjugated anti-rabbit or anti-mouse secondary antibody. Western blots were exposed to chemiluminescent reagents (NCM Biotec, Suzhou, China, P10060) and analyzed by an imager (Tanon, Shanghai, China, 5200Multi). The following primary antibodies were used: anti-Flag (Sigma-Aldrich, St. Louis, MI, USA, F3165), anti-p-RIP (Cell Signaling, Danvers, MA, USA,, 65746), anti-HSP65 (Santa Cruz, TX, USA, sc-58170), anti-GFP (Huaxingbio, Beijing, China HX1824), anti-His (Huaxingbio, HX1822), and anti-GAPDH-HRP (Bioworld, St. Louis Park, MN, USA, MB001H). Anti-Pvc16 was generated from immunized rabbit serum using specific synthesized oligopeptides (Genscript, Piscataway, NJ, USA).

### 4.4. PVC Purification

The purification protocol for the PVC was adapted from the previously published literature [37]. Briefly, the LysR-producing plasmid pBR322 was transformed into the *E. coli* EPI300 strain, which already harbors the plasmid pRK404-PVC responsible for expressing PVC structural genes. When cargo protein expression was required, an additional pBBRN-pdp1N50 plasmid carrying the relevant genes was co-transformed into the same strain. Cultures were grown overnight in 200 mL of LB broth at 30 °C for 16 h. Bacterial pellets were harvested and resuspended in 30 mL of lysis buffer P [25 mM Tris-HCl (pH 7.4), 140 mM NaCl, and 3 mM KCl, supplemented with deoxyribonuclease I (50 µg/mL), lysozyme (200 µg/mL), 0.5% Triton X-100, 5 mM MgCl2, and a 1× protease inhibitor cocktail (MedChemExpress, HY-K0010)]. The suspension was incubated at 37 °C for 30 min to facilitate cell lysis. Following lysis, cell debris was removed by centrifugation at 14,000 rpm for 10 min at room temperature. The clarified supernatant was then ultracentrifuged at 150,000× *g* for 60 min at 4 °C to pellet the protein complexes. The resulting pellet was gently resuspended in 1 mL of sterile phosphate-buffered saline (PBS). A second round of centrifugation at 14,000 rpm for 10 min at 4 °C was performed to remove residual contaminants. Subsequently, the supernatant was subjected to a second ultracentrifugation step at 150,000× *g* for 60 min at 4 °C to further purify the PVC. Finally, the purified pellet was resuspended in 200 µL of ice-cold PBS and clarified by a final centrifugation step at 14,000 rpm for 10 min at 4 °C. The supernatant containing the highly purified PVC was aliquoted and stored at 4 °C for short-term use.

### 4.5. Cell Transfection

When the confluence of HEK293T cells reached 70%, the plasmids pEGFP-C1, pEGFPC1-MmTG, pEGFPC1-C159S, and pEGFPC1-MAB 4306 were transiently transfected into HEK293T cells at a concentration of 0.1 μg/well (96-well plate) or 0.5 μg/well (24-well plate) using the PolyJet transfection reagent (SignaGen, Frederick, MD, USA, SL100688) according to the manufacturer’s instructions. After 24 h of transfection, the transfection efficiency was determined by observing the expression of green fluorescence under a fluorescence microscope for subsequent experiments.

### 4.6. Cell Viability Assay

Cell viability was determined using the Cell Counting Kit-8 (MedChemExpress, HY-K0301) and CellTiter-Lumi™ Luminescent Cell Viability Assay Kit (ATP content analysis, Beyotime, Shanghai, China, C0065M) according to the manufacturer’s instructions. Each test should have at least three parallel groups, and each experiment should be repeated at least three times.

### 4.7. In Vitro Infection with Recombinant M. smegmatis

J774A.1 and THP-1 cells were seeded at 1 × 10^5^ cells/well in 96-well tissue culture plates and cultured without any antibiotics, and the cells were then infected with Ms_Vec and Ms_MmTG at an MOI of 5 or 10. Four hours after infection, the infected cells were washed using PBS, and gentamicin (100 μg/mL) was used to kill the bacteria outside of the macrophages after four hours of infection. Then, cells were washed once with PBS and culturing was continued for 18 h using an antibiotic-free medium. The cell morphology was observed under a microscope and the cell viability was tested.

### 4.8. Bioinformatics and Statistical Analysis

All sequences were obtained from the NCBI and UniProt. Protein sequence alignment and phylogenetic tree construction were performed using DNAMAN and MEGA12. The phylogenetic tree was visualized using FigTree v1.4.4. Protein structure prediction was conducted through Alpha Fold3.0, and protein structure comparison and visualization were carried out using PyMOL software. Data processing was carried out with Graphpad, and data were analyzed by a *t*-test and analysis of variance for significance.

## 5. Conclusions

This study reports that MmTG exhibits significant cytotoxicity toward eukaryotic host cells. Cys159 is a conserved active site with its cytotoxicity being attenuated upon site-directed mutagenesis. Bioinformatics analysis reveals that TGases are widely distributed and highly conserved among Mycobacterium. Studies have shown that both MmTG and its homolog can induce the phosphorylation of RIPK1, suggesting that this widely conserved MmTG in Mycobacterium may share similar cytotoxic mechanisms. These findings provide valuable insights for further investigation of Mycobacterium pathogenesis.

## Data Availability

The original contributions presented in this study are included in this article and the Supplementary Materials. Further inquiries can be directed to the corresponding authors.

## Supplementary Materials

The following supporting information can be downloaded at: https://www.mdpi.com/article/10.3390/molecules30102251/s1, Figure S1. The homologous protein MAB_4306 exhibits cytotoxicity and functions through the conserved C159; Figure S2. The transglutaminases from eight conditionally pathogenic *Mycobacterium* species exhibit evolutionary conservation and a highly conserved cysteine active site; Figure S3. Restricted digestion of plasmid vectors and gene fragment cloning; File S1: Table S1. Protein sequences of eight transglutaminases derived from different bacterial species; Table S2. Protein sequences of eight transglutaminases with opportunistic pathogens to humans and animals; Table S3. Primers used in the study; File S2: Protein sequences from 1000 different bacterial species.
