# Supplementary material for: Identification of the Cytotoxic Transglutaminase from Mycobacterium spp. That Is Involved in RIPK1 Activation
Source: Molecules. 2025 May 21;30(10):2251. doi: 10.3390/molecules30102251 (PMC12113844; doi:10.3390/molecules30102251)
Supplement: Supplementary file 1 [file molecules-30-02251-s001.zip › Molecules SI.pdf]

molecules

MDPI

Supplementary material

Identification of the Cytotoxic Transglutaminase from *Mycobacterium* spp. That Is Involved in RIPK1 Activation

Xinting Zhang †, Yikai Zhang †, Xiao Feng, Yueying Wang, Si-Shang Li, Mei-Yi Yan, Yi-Cheng Sun, Qi Jin * and Feng Jiang *

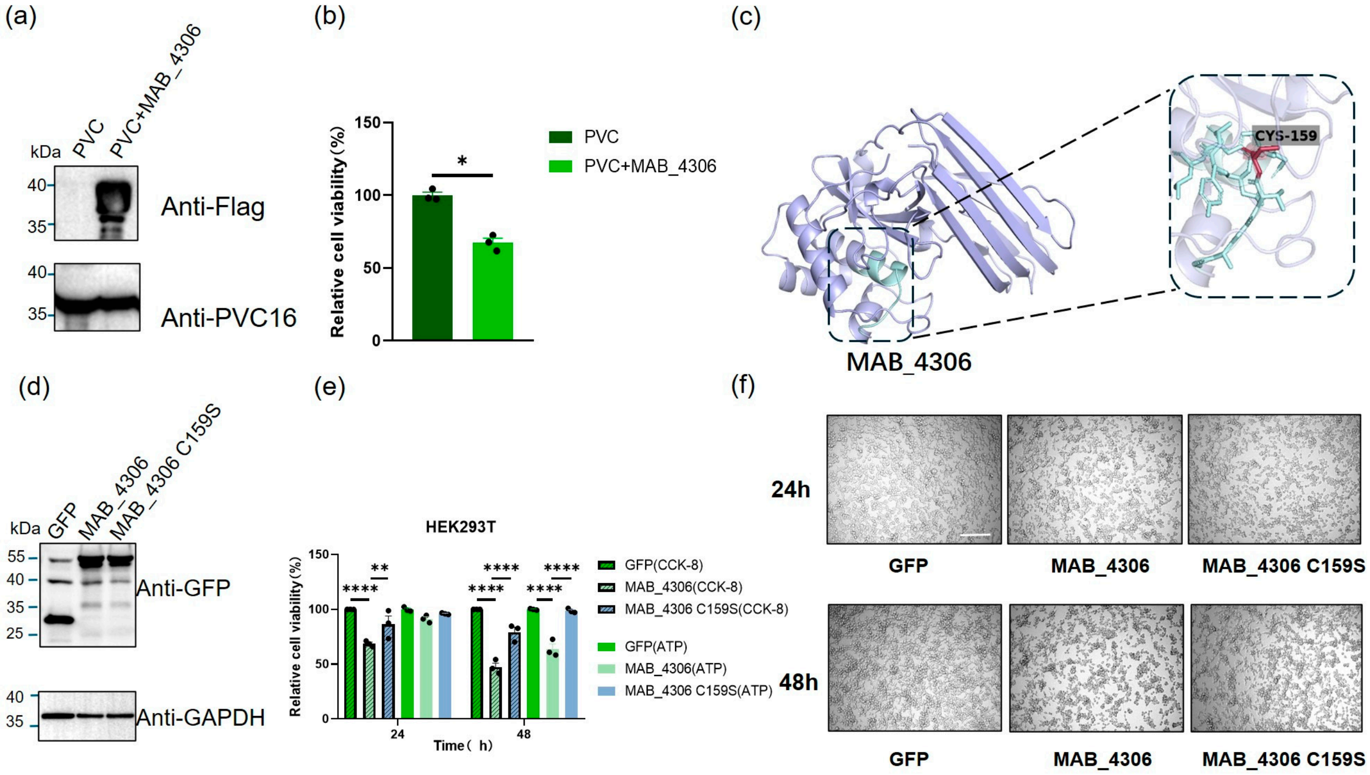

Figure S1. The homologous protein MAB_4306 exhibits cytotoxicity and functions through the conserved C159. (**a**), Loading of MAB_4306 was detected by Western blot. Pvc16 was used as a loading control. (**b**), Cell viability of J774A.1 cells was assessed using the CCK-8 at 24 hours after delivery of MAB_4306 by PVC. (**c**), 3D model of MAB_4306 module. Sequences of the MAB_4306 were analyzed by AlphaFold3 and the C159 key residue was highlighted. (**d**), Western blot was used to detect the transient expression of MAB_4306 and C159S in HEK293T cells. (**e**), Cell viability of HEK293T cells was assessed using the CCK-8 (with slashes) and ATP content analysis (no slashes) at 24 hours and 48 hours post-transient expression of MAB_4306 and C159S, respectively. (**) $p < 0.01$; (****) $p < 0.0001$. (**f**), Cell morphology following 24-hour and 48-hour transient expression of MAB_4306 and C159S in HEK293T cells. Scale bars, 200 μm.

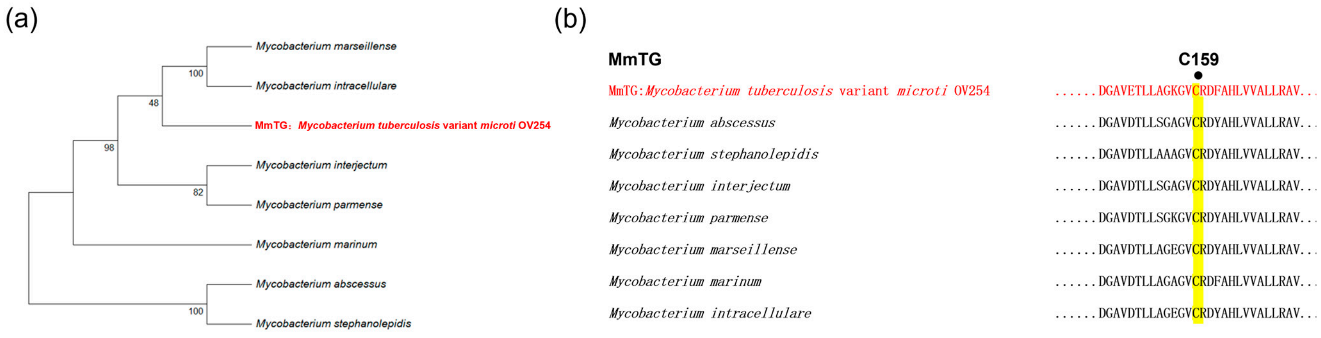

Figure S2. The transglutaminases from eight conditionally pathogenic *Mycobacterium* species exhibit evolutionary conservation and a highly conserved cysteine active site.(**a**) To analyze the evolutionary relationships of transglutaminases in eight conditionally pathogenic *Mycobacterium* species infecting humans and animals, a phylogenetic tree was constructed using the Neighbor-Joining method. (**b**) The cysteine residue at position C159 of MmTG is highly conserved among the eight conditionally pathogenic Mycobacterium species.

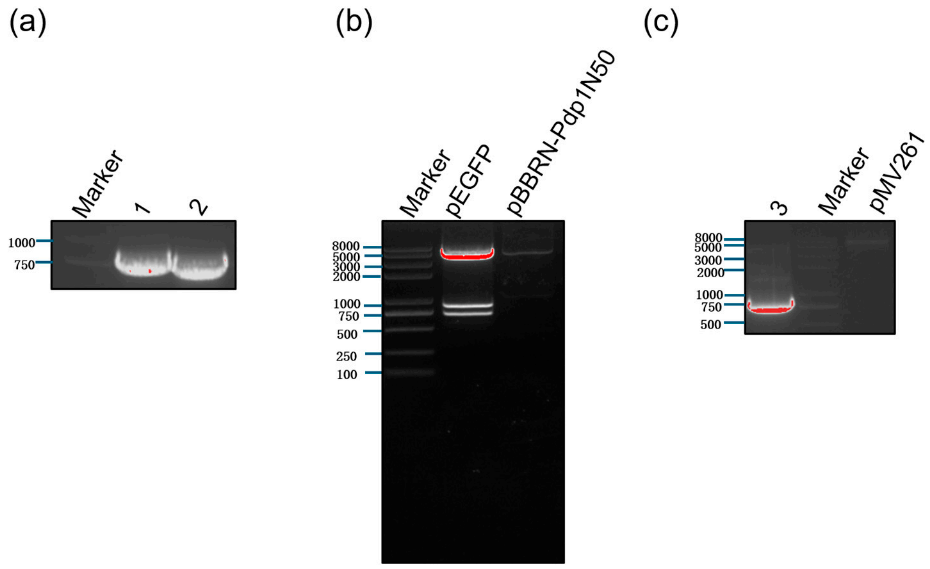

Figure S3. Restriction digestion of plasmid vectors and gene Fragment cloning. (**a**) PCR amplification of MmTG gene fragments. Lane 1: PCR amplification of MmTG using primers designed with homologous arms corresponding to the restriction sites of the pEGFP-C1 plasmid vector, followed by agarose gel electrophoresis. Lane 2: PCR amplification of MmTG using primers containing homologous arms matching the restriction sites of the pBBRN-Pdp1N50 plasmid vector, followed by agarose gel electrophoresis. (**b**) The pEGFP-C1 plasmid vector was double-digested with BglII and SalI, followed by agarose gel electrophoresis to assess digestion efficiency and recover the target band. The pBBRN-Pdp1N50 plasmid vector was similarly digested with BamHI and HindIII, and the digested products were analyzed and the desired fragments recovered. (**c**) The pMV261 plasmid vector was digested with BamHI and HindIII, then subjected to agarose gel electrophoresis to verify the digestion and recover the target fragment. Lane 3: PCR amplification of MmTG using primers designed with homologous arms corresponding to the restriction sites of the pMV261 plasmid vector, followed by agarose gel electrophoresis.
